# Ethnobotanical study on ritual plants used by Hani people in Yunnan, China

**DOI:** 10.1186/s13002-024-00659-y

**Published:** 2024-02-13

**Authors:** Xueyu Ma, Dan Luo, Yong Xiong, Caiwen Huang, Ganpeng Li

**Affiliations:** 1 Yunnan Institute for Ethnic Studies/School of Ethnology and History, Yunnan Minzu University, Kunming, 650504 Yunnan China; 2 School of Ethnomedicine and Ethnopharmacy, Yunnan Minzu University, Kunming, 650504 Yunnan China; 3 Institute of Ethnology, Yunnan Academy of Social Sciences, Kunming, 650034 Yunnan China

**Keywords:** Hani people, Ethnobotany, Ritual plants, Traditional rituals, Cultural connotation

## Abstract

**Background:**

The Hani people, who reside in Yuanyang County, Honghe Hani and Yi Autonomous Prefecture, Yunnan Province, rely on rice terrace farming as their primary livelihood. They utilize plants in various traditional ritual practices. The Hani people have categorized the value of plants based on their natural attributes and have refined the ways of using different plants in specific rituals through practical observations and experiences derived from their agricultural culture. Although the plants used in these rituals hold significant cultural value, they have yet to be studied from the perspective of ethnobotany. This study aims to approach the ritual plants using ethnobotanical methods.

**Methods:**

Ethnobotanical fieldwork was conducted in 10 villages in Yuanyang County between 2021 and 2023. Data were collected from the local Hani people through semi-structured interviews and participatory observations and 41 informants were interviewed during the field investigations. The frequency of citation (FC) and relative frequency of citation (RFC) were utilized to evaluate the relative importance of ritual plants among the local communities.

**Results:**

A total of 36 plant species, belonging to 18 families and 34 genera, were recorded as being used in 11 ritual practices by the Hani people. Rosaceae, Poaceae, and Fabaceae were found to have the highest number of species. Most of the ritual plants used by the Hani people were collected from the wild. FC and RFC analysis showed that the preferred plants for Hani rituals were *Rhus chinensis* Mill, *Oryza sativa* L., *Phyllostachys sulphurea* (Carr.) A. et C. Riv. and *Musa basjoo* Siebold & Zucc. ex Iinuma. The 11 rituals are all centered around the performance of people, crops and livestock. The Hani people use plants in different rituals mainly based on their biological attributes.

**Conclusions:**

Many rituals of the Hani people are closely related to their production and livelihood, and the plants used in these rituals are deeply rooted in Hani’s traditional ecological knowledge and beliefs. The Hani people’s reverence for nature, respect for life, gratitude towards ancestors, and seeking blessings and disaster prevention for their families, crops, and livestock are all reflected in these rituals and their utilization of ritual plants. The Hani people showcase their agricultural culture in the Honghe Hani Rice Terraces through plant-based ritual performances. Studying ritual plants in the core area of the Hani Rice Terraces is of great significance for protecting the Hani Terrace farming culture. In the future, it is essential to pay more attention to the role of traditional knowledge in biodiversity conservation.

## Background

The relationship between humans and plants has always been the scope of ethnobotany [1]. After more than 100 years of development, ethnobotanical research has expanded to many aspects of people’s lives. The use value of plants is inherent in their natural attributes, while how plants are utilized is determined by culture [2]. Local communities have long maintained a balance with the ecological environment based on local knowledge. Geertz posits that local knowledge is ontological, something natural and inherent in the local culture [3]. In Arne Kalland’s three-tiered interpretation of local knowledge, it is described as “experiential knowledge involving the recognition of flora and fauna and the purposes and methods of their utilization.” [4] Traditional ecological knowledge of various ethnic groups is how local people perceive, respond to, and contemplate the world, emphasizing a spatial holistic view that recognizes the interconnectedness of all things in the universe. It is also an experiential and practical capacity that guides life and facilitates the process of dialogue and communication between individuals and nature [5]. In recent years, research on local knowledge has yielded abundant results, and research perspectives have become more diverse [6].

Turner posits that ritual refers to formalized behavior when people rely on belief in mystical substances or powers without recourse to technical procedures [7]. The ritual aims to exorcise evil spirits, ward off disasters, and protect the balance of all things in the three realms of space [8]. 28 plant taxa among Bukovinian Hutsuls and 58 plant taxa among inhabitants in Roztochya were used in 7 religious festivals; these plants were mainly used in bouquets, but also for decorating churches and houses or fruit baskets [9]. The Naxi people use 32 species of plants in ritual practices, mainly for incense and decoration [10]. In Shaxi, the Bai people use 17 ceremonial plants to burn incense to communicate with ancestors, ghosts and spirits, and in some cases to enhance self-awareness [11]. The Liangshan Yi people use a variety of plants in traditional folk customs, and their cultural significance is mainly reflected in three aspects: exorcism, reproductive worship and ancestor worship [12]. The Akha people regard fleagrass (*Adenosma buchneroidest*) as a tribal symbol and a gift of love [13]. Indigenous people believe that ceremonial plants can be utilized for ritual healing purposes [14, 15], as well as incense or ornaments used for communication with deities [16].

The ecological worldview of the Hani people is partially reflected in their understanding and utilization of plants. Current scholarly research on the relationship between the Hani people and plants primarily focuses on medical, symbolic, and ritual aspects. Plants can alleviate the pain caused by the dangers of the “unstable” environment where the Akha people live, particularly during practical activities in fields and forests [17]. The Akha people are a branch historically differentiated from the Hani ethnic group, and they share close cultural and customary similarities with the Hani people [18]. The Hani people’s “Angma” is a protective deity ensuring the village’s safety. Her dwelling is within a forest above the village, marked by a specific tree. Legends and ritual processes among different Hani communities share similarities in their worship of the tree as the village’s guardian deity [19]. During rituals, plants are directly worshipped by the Hani people. The association between plants and the Hani people and their culture is primarily expressed through symbolic actions and the significance of symbolic symbols in the ritual process [20]. In addition to meeting the Hani people’s daily material needs, plants also possess symbolic meanings in religious and psychological aspects, embodying the cultural significance of the Hani people [21]. The Hani people’s material life and spiritual beliefs are closely related to plants.

In 2010, the Honghe Hani Rice Terraces were listed as a Globally Important Agricultural Heritage System (GIAHS) by the Food and Agriculture Organization of the United Nations (FAO), and in June 2013 they were listed as a World Cultural Landscape Heritage by United Nations Educational, Scientific and Cultural Organization (UNESCO). The Honghe Hani Rice Terraces are concentrated and widely distributed in Yuanyang County on the southern slope of the Ailao Mountains in the south of Yunnan Province. The Hani people who have lived in Yuanyang County for generations, rely primarily on terraced rice cultivation as their main livelihood. Based on their plant worship and folk beliefs, various traditional rituals have emerged surrounding their production and daily life, incorporating plants in various forms across these rituals. The laws of life exist widely in nature; wherever there is life, there are laws [22]. The Hani people use plants to perform rituals and express their will through rituals while also following their natural laws. Plants are crucial mediators, establishing a transcendent connection with nature during the ritual. Depending on the occasion, the Hani people select different plants for different rituals, mainly based on the characteristics of their biological properties, such as their scent, shape, color, lifestyle, and ease of collection. Abundant plants provide energy, vitamins, and other nutrients for the Hani people but also play an essential role in various rituals. Understanding and utilizing plants is an essential core of the Hani people’s ecological practices; the Hani people also preserve their traditions through ritual practices that are repeatedly performed.

To our knowledge, no previous ethnobotanical research records exist on ritual plants within the Hani terraced agricultural ecosystem. Therefore, in various traditional rituals, we investigated the ritual plants used by the Hani people, who rely on terrace farming as their primary livelihood. This study fills a gap in the existing knowledge. The Hani people’s ritual performances are specific expressions and important carriers of their traditional culture. In addition to documenting the ritual plants, we meticulously recorded and organized relevant information about the rituals. Such research holds significant importance in globalization and digitalization, as it contributes to preserving traditional ecological knowledge among the Hani people. Many Hani ritual experts or folk inheritors face the challenge of a lack of successors, and the traditional knowledge of ritual plants is under a destructive threat. Knowledge of ritual practices and plants can be supportive material for discussing how folk beliefs can promote conservation efforts.

## Methods

### Study area

**Fig. 1:**
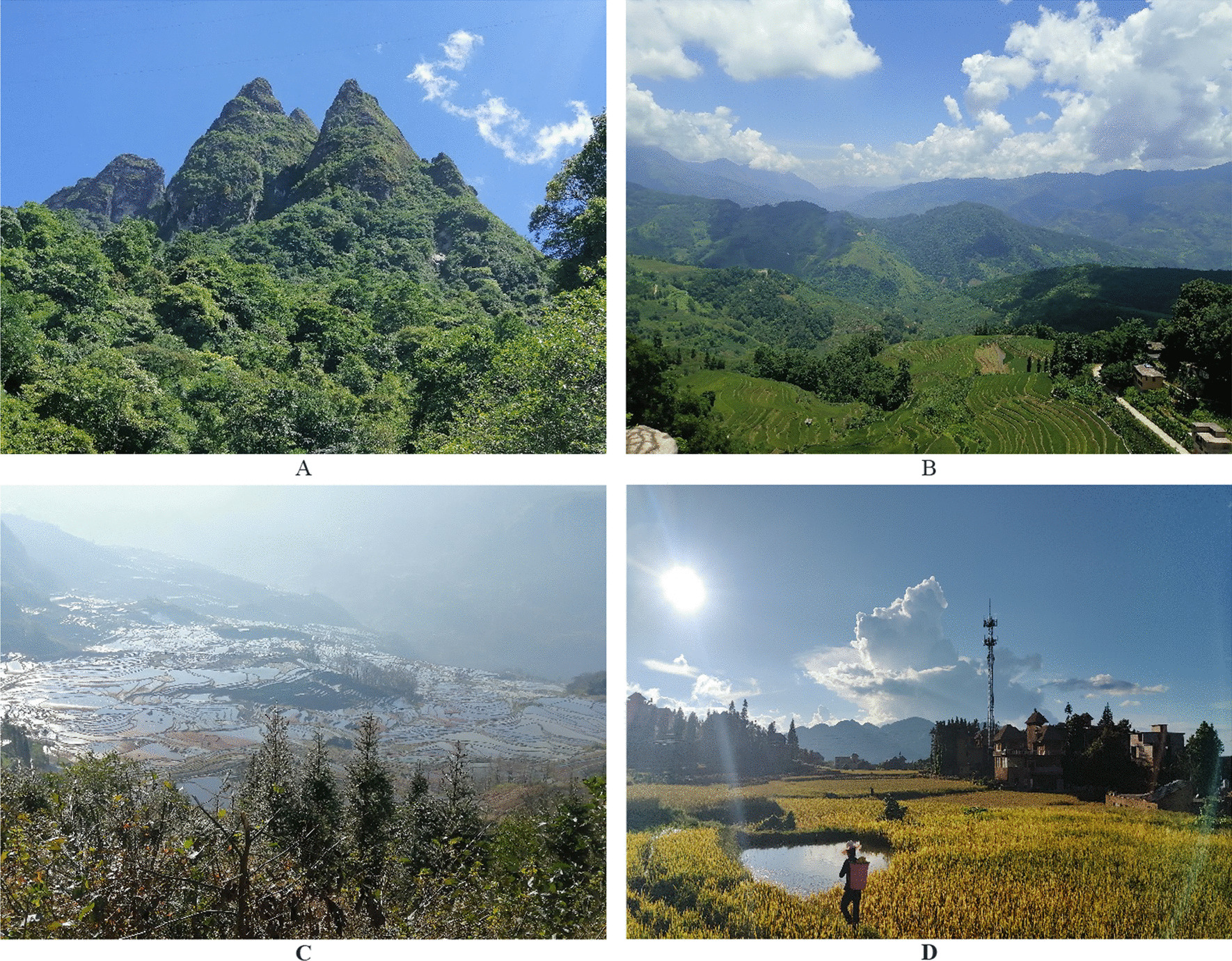
**A** Wuzhi Mountains above the villages studied; **B** Summer at Hani Rice Terraces in Duo Jiao village; **C** Terraces in the morning light in A ZheKe village; **D** Golden terraces in Da YuTang village

The research area of this study is situated in Yuanyang County, Honghe Hani and Yi Autonomous Prefecture, Yunnan Province, China. Yuanyang County is located in the southern part of Yunnan Province, along the southern bank of the Honghe River, and in the southern segment of the Ailao Mountains. Geographically, it lies between 102°27ʹ–103°13ʹ east longitude and 22°49ʹ–23°19ʹ north latitude. The region falls within the southwestern part of China’s Yunnan-Guizhou Plateau, characterized by a low latitude, high altitude, and a monsoonal climate. The area exhibits a prominent vertical stratification of mountainous terrain, contributing to a complex three-dimensional climatic pattern. Yuanyang County is situated in a region of high altitude and low latitude, characterized by undulating mountain ranges, crisscrossing valleys, and continuous mountainous terrain without any flat plains. The highest point is the summit of Baiyanzi Mountain, located in the Dongguanyinshan Peak within Ganiang Township, with an elevation of 2939.6 m above sea level. The lowest point is at the exit of the Honghe River within Fengchunling Township, with an elevation of 144 m above sea level [23] (Fig. 1).

**Fig. 2:**
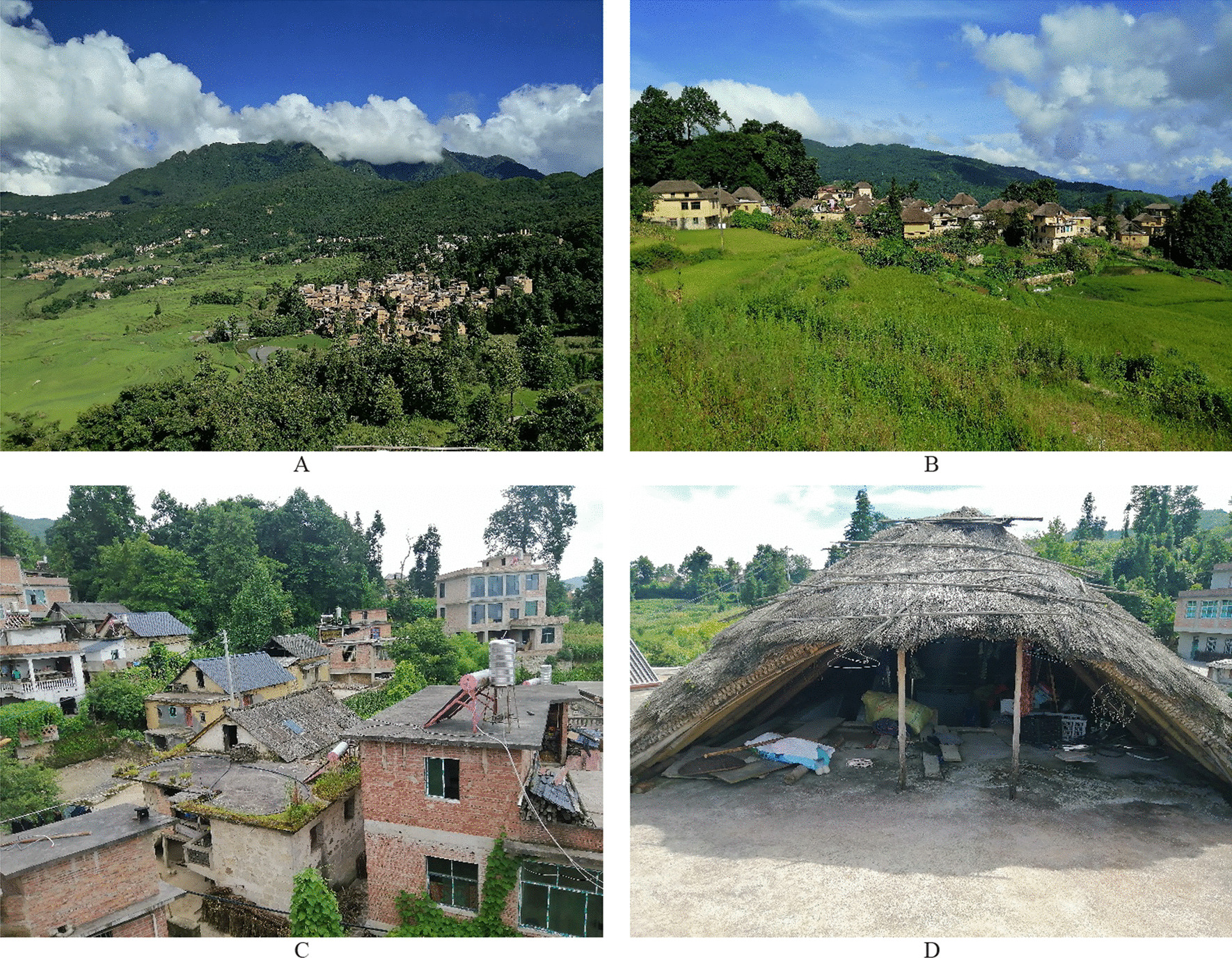
**A** The four isomorphisms of the Hani terraces; **B** Mushroom-like house in A ZheKe; **C** The reinforced concrete houses in Hani Village; **D** Mushroom-like roof in Ai Cun

Yuanyang County has seven ethnic groups: Hani, Yi, Dai, Zhuang, Yao, Miao, and Han. According to relevant records, the Hani population in Yuanyang County is 254,997, accounting for 55.5% of the total population [23]. Hani and Yi have settled in the upper levels, with Hani, Zhuang and Yao at the middle levels, and Dai and Yi at the lower levels of the watershed [24]. The Hani people are known for their traditional and distinctive architectural style called “Mushroom houses”. The term “Mushroom house” is derived from its resemblance to the shape of a mushroom. Mushroom houses typically consist of three levels: the first is primarily used for storage and livestock, the second serves as living space for people, and the third is used for grain storage and drying. However, with the development of the economy and the changes of the times, most mushroom houses have gradually been replaced by reinforced concrete buildings (Fig. 2).

The Hani people have a long history, many branches, and diverse cultures. They have no traditional written language but rich folk religious beliefs. The Hani people are mainly distributed in the Yunnan Province of China, Vietnam, Laos, Myanmar, and the mountainous areas of northern Thailand in Southeast Asia. The Hani people in China mainly live in the “Three Rivers and Two Mountains” area of Yunnan, namely the Red River, Balbian River, Lancang River, Ailao Mountains, and Wuliang Mountains [25]. This article mainly discusses those Hani people who reside on the southern section of Ailao Mountains on the south bank of the Red River, have lived in Yuanyang County for generations, and rely on terraced rice farming as their primary way of livelihood rather than all Hani people in China. As one of the leading creators of the landscape form and terrace culture of the Hani Rice Terraces, a world cultural heritage, and one of the main ethnic groups living in the core area of the Hani Rice Terraces, the Hani people predominantly follow their traditional folk religion. Plants from the natural world are indispensable and essential elements in the traditional rituals of the Hani people. Plants used in various rituals are collectively called “ritual plants” [26]. We found that the Hani people select different plants for various rituals. Specific rituals require a distinct set of plants with unique functions and meanings. The utilitarian value of plants as local knowledge is perpetuated through intergenerational transmission within the traditional social structure of the Hani community. The Hani people’s worldview, philosophy of life, values, perception of illness, ecological understanding, and cosmology are all reflected through their use of plants. The Hani people and plants mutually influence and shape each other, fostering a symbiotic relationship. Humans interact with nature and biodiversity daily, although many may not fully grasp the diverse ways in which the natural world permeates their lives [27]. These interactions have forged a close and mutually beneficial bond between the Hani people and plants. Hani people’s needs, selection, and utilization of plants contribute to their propagation, while the material and spiritual values provided by plants ensure the continuity of human life.

### Identification of research sites and informants

Firstly, we conducted extensive long-term field investigations and found that the Aichun Administrative Village in Xinjie Town, Yuanyang County, Honghe Hani and Yi Autonomous Prefecture in Yunnan Province, China, is located in the core area of Hani terraced fields, where the terraced culture is well-preserved. The six natural villages under its jurisdiction, namely Da YuTang, Ha DanPu, A ZheKe, Niu LuoPu, Ai Cun, and Yan ZiJiao, are regions where ritual experts are relatively concentrated. Various traditional rituals are still well-preserved in these villages. The remaining four villages, Sheng Cun, Da ZhongQiao, Duo Jiao, and Qi Zuo, were identified through a snowball sampling method used for information gathering. Secondly, in many Hani villages where terrace rice farming is the main livelihood, traditional folk religious figures are no longer present, and many festivals and rituals have been simplified or even disappeared.

**Fig. 3:**
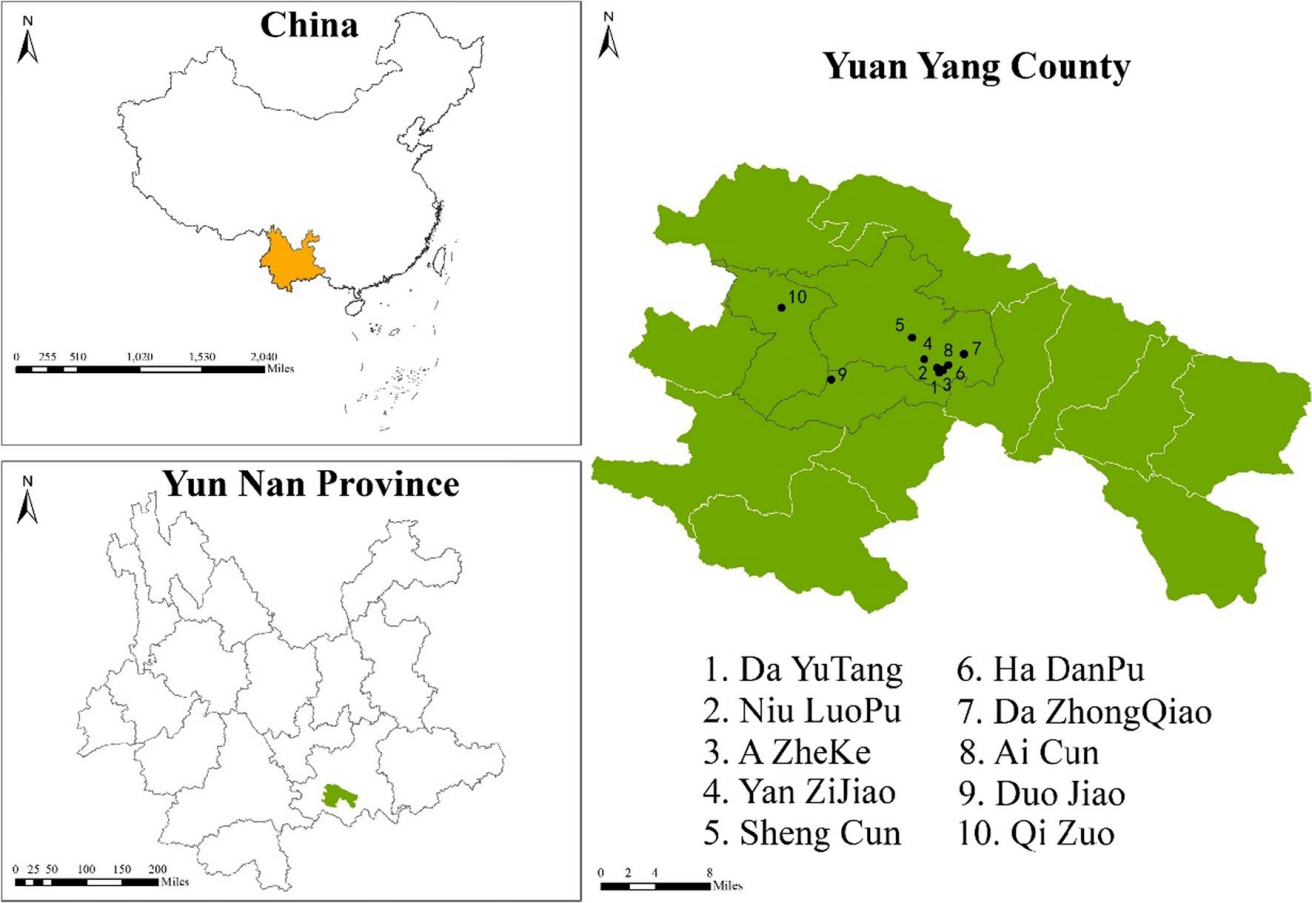
Map of the study area

**Fig. 4:**
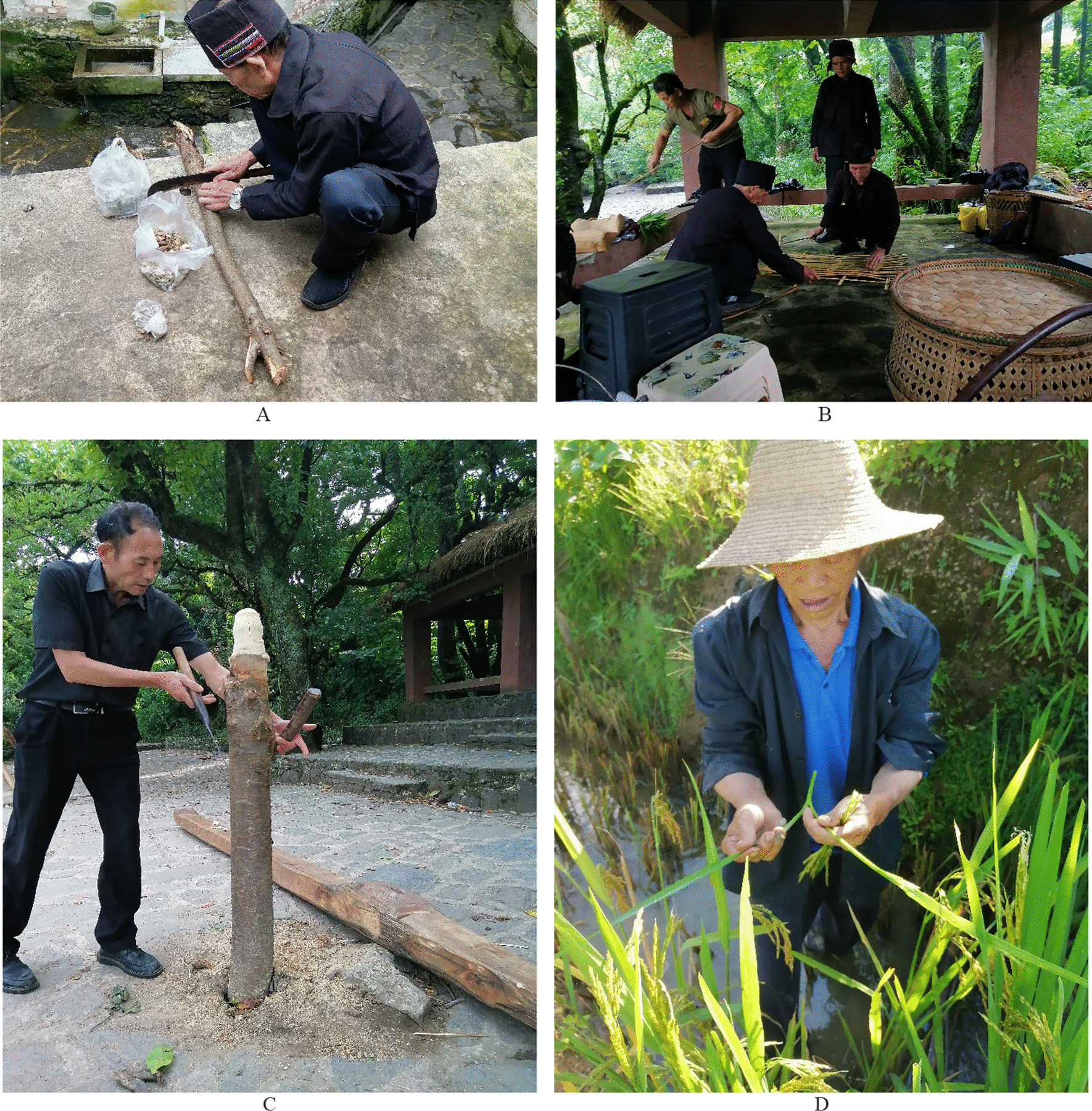
**A** Beima prepares sacrifices for the ritual of asking for peace “Hasaza”; **B** Four Migus use *Chimonobambusa pachystachys* Hsuch et W. P. Zhang to make sacrificial altar “Boge” on Kuzhazha Festival; **C** Migu makes the Moqiu pillars on Kuzhazha Festival; **D** Migu carefully selects the rice ears used for the Xinmijie Festival

From October 2021 to December 2023, we collected ethnobotanical data in ten villages of the study area (Fig. 3). A total of 41 informants in the study, all of whom belong to the Hani people, have been interviewed. The selection of these informants was based on the snowball sampling method (Fig. 4). We recorded the demographic information of the informants, such as age, gender, and occupation during the interview process. The 41 informants are not ordinary villagers; they are ritual experts, and ritual participants. Among them, 11 possessed expertise in Hani ritual practices and were recognized as ritual experts, referred to as “Migu” or “Beima” in the Hani language; they often served as ritual hosts and/or collectors of ritual plants. Ritual participants included individuals who received the rituals and/or collectors of ritual plants. Informants’ ages ranged from 25 to 84 years old, with the majority (87.80%) over 40. Informants were mainly male. Among them, more than 85.37% (35 respondents) were local farmers whose primary livelihoods were terraced farming, four were civil servants, and two were college students (Table 1).

**Table 1:** Demographic features of informants

Demographic features	Number	Proportion (%)
*Sex*		
Male	34	82.93
Female	7	17.07
*Age*		
20–30	2	4.88
31–40	3	7.32
41–50	14	34.15
51–60	17	41.46
61 and above	5	12.2
*Relationship with ritual*		
Ritual experts	11	26.83
Ritual participants	30	73.17

This research adhered to international ethical guidelines, ensuring informed consent was obtained from each participant before their interview. Prior to conducting the interviews, verbal consent was obtained from each individual.

### Methods for acquiring ritual plants and related knowledge

Firstly, we initiated our investigation by actively participating in observing rituals. Due to the extensive duration of the field research and our close interaction with the villagers, including living and working together, we could personally experience the ritual scenes and observe the entire process. During the rituals, we witnessed the Hani people use specific plants or plant combinations, enabling us to record detailed plant information. Secondly, during the long-term field investigation, the authors had numerous opportunities to conduct in-depth interviews with individuals who had significant involvement with ritual plants, i.e. ritual experts and ritual participants.

**Fig. 5:**
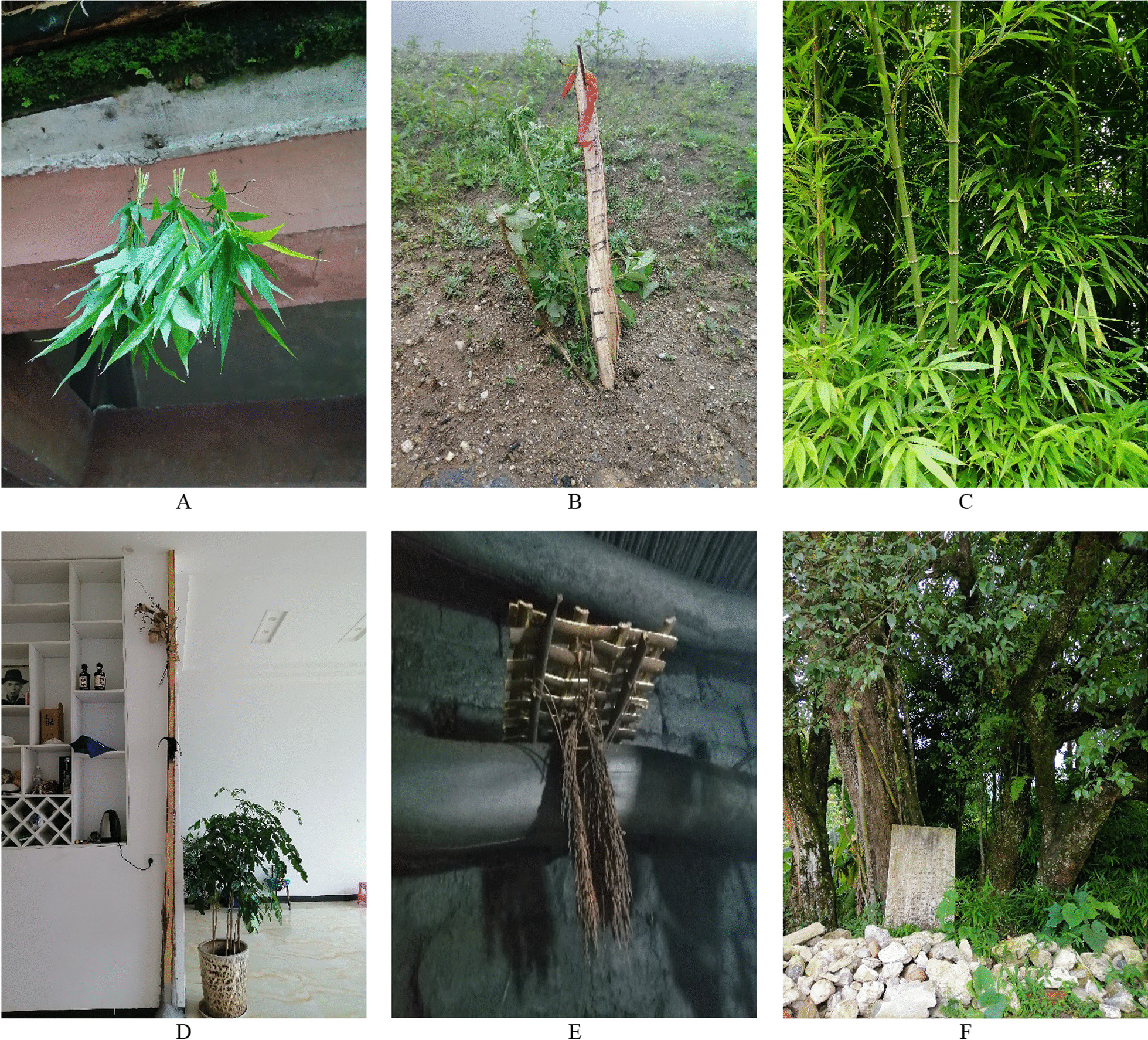
**A** *Microstegium ciliatum* (Trin.) A. Camus hanging in front of the “Moqiu” house during the Kuzhazha Festival;** B** Four plants (*Artemisia caruifolia* Buch.-Ham. ex Roxb; *Capsicum annuum* L.; *Rhus chinensis* Mill; *Rubus ellipticus var. obcordatus* (Franch.) Focke) used in ritual to drive away swine fever;** C** *Chimonobambusa pachystachys* Hsuch et W. P. Zhang;** D** *Cunninghamia lanceolata* (Lamb.) Hook used in the ritual of erecting a central pillar;** E** Sacrificial altar “Boge” made of *Chimonobambusa pachystachys* Hsuch et W. P. Zhang; **F “**ShanZhabei” protected by *Pyrus betulifolia* Bunge and *Crataegus pinnatifida* Bunge, which were planted in the ritual of erecting the stone tablets of merit

We employed semi-structured interviews to gather information about ritual plants from the informants. We conducted interviews in the local Honghe dialect, not the Hani language. All the participants were proficient in the local Honghe dialect. Before starting the interviews, we conducted preliminary inquiries to identify potential interviewees. For each plant discussed, we accompanied the interviewees to the field to collect corresponding specimens, ensuring the accuracy of the research findings (Fig. 5).

**Fig. 6:**
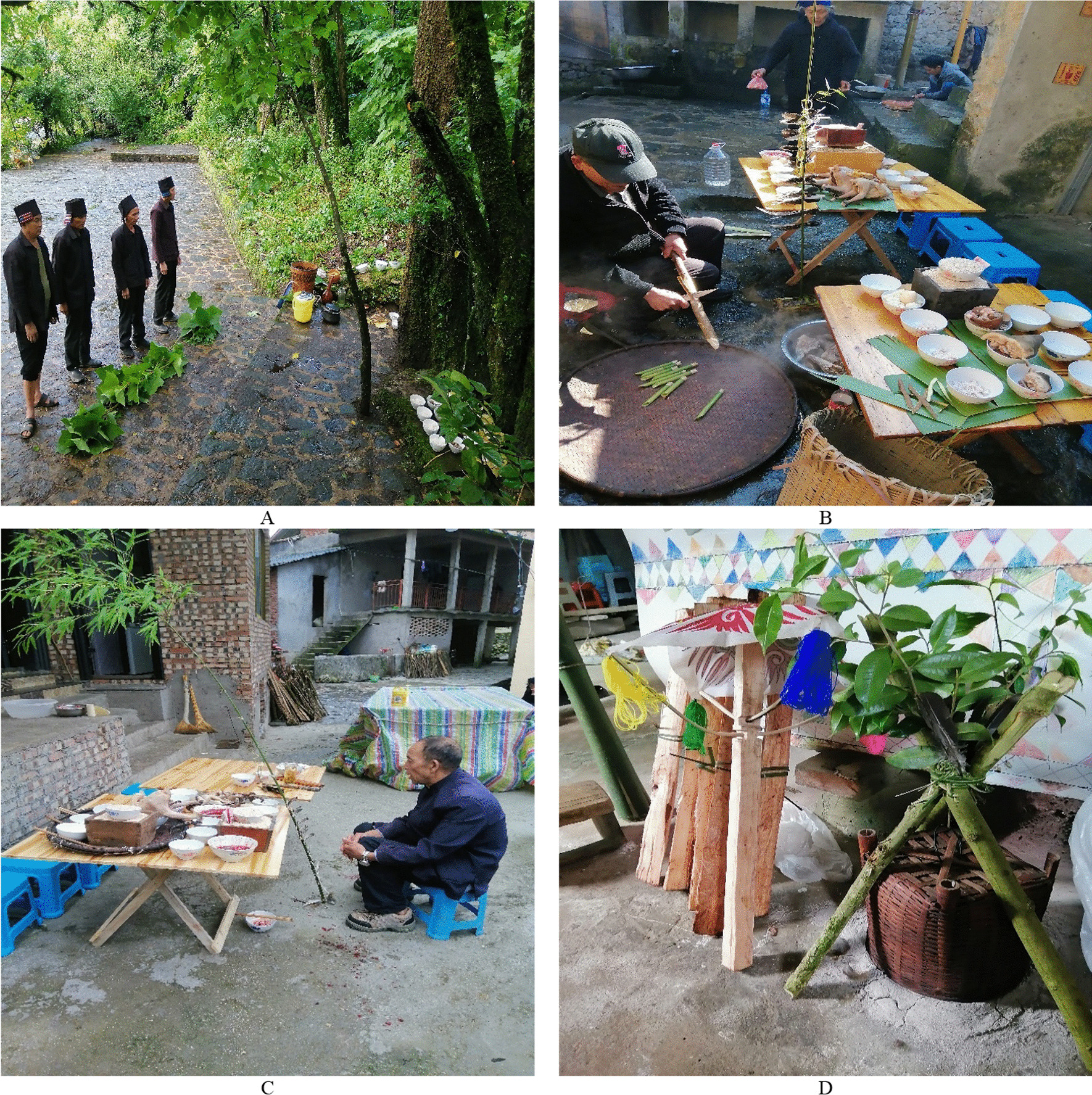
**A** Four Migus worship the sacred tree during the Kuzhazha Festival**; B** Beima prepares plants for the ritual of calling sb’s soul “Ahuihuisuohong”**; C** Beima is reciting the sacrificial words to the ancestral spirit made by *Phyllostachys sulphurea* (Carr.) A. et C. Riv. in purification ritual “Dekayaza”**; D** “Ghost umbrellas” made by in *Alnus nepalensis* D. Don and *Eurya nitida* Korthals in funeral

During the interview, we asked the respondents three questions: Which plants do you choose when performing these rituals? How to use these plants? Why do you select this particular species or group of plants and what are the stories behind using these plants? We recorded detailed information about the process of each ritual and the specific use of plants through participant observation. Voucher specimens were deposited in the herbarium of the School of Ethnomedicine and Ethnopharmacy, Yunnan Minzu University (Fig. 6).

### Data analysis

First, according to the semi-structured interviews with three guiding questions, we subsequently organized, classified, and cataloged all collected plant information, including scientific name, Chinese name, Hani name, family, habit, and habitat. We utilized FC and RFC analyses to identify the frequently mentioned plants and summarized the number of rituals each plant were used (Table 2). Secondly, we systematically sorted the data according to the complete ritual process conducted by the Hani people, including the time, location, participants, causes, functions, and significance of each ritual. We provided detailed descriptions of the ritual information (Table 3) and how the plants were prepared and utilized within each ritual (Table 4). Finally, we analyzed data to explore why specific plants were used in the rituals (Table 5). All the data were stored and analyzed in Microsoft Excel 2017.

**Table 2:** List of ritual plants used by Hani People

No	Scientific name	Chinese name	Hani name	Hani name (phonetic)	Family	Habit	Habitat	FC	RFC	Number of rituals used	Specimen number
1	*Acer sinense *Pax	Zhonghuaqi 中华槭	aoqhaceiqkavq	Ehacega	Sapindaceae	Tree	Wild	4	0.10	1	HNYS-001
2	*Alnus nepalensis* D. Don	Qimu 桤木	haqnivlssaq	Haniran	Betulaceae	Tree	Wild and cultivated	34	0.83	2	HNYS-002
3	*Amomum tsaoko* Crevost et Lemarie	Caoguo 草果	meilhhoqalzaq	Meiwuza	Zingiberaceae	Herb	Cultivated	25	0.61	1	HNYS-003
4	*Artemisia caruifolia* Buch.-Ham. ex Roxb	Qinghao青蒿	eihaq	Aiha	Asteraceae	Herb	Wild	31	0.76	1	HNYS-004
5	*Bambusa emeiensis* L. C. Chia & H. L. Fung	Cizhu 慈竹	haqbol	Habo	Poaceae	Herb	Wild	32	0.78	2	HNYS-009
6	*Capsicum annuum* L	Lajiao 辣椒	laqpil	Lapi	Solanaceae	Herb	Cultivated	30	0.73	1	HNYS-012
7	*Castanopsis chinensis* (Sprengel) Hance	Zhui 锥	qiqqeilssaq	Qiqianran	Fagaceae	Tree	Wild	33	0.80	2	HNYS-014
8	*Celtis tetrandra* Roxb	Siruipu 四蕊朴	hoqbuqssaq	Huoburan	Cannabaceae	Tree	Wild	27	0.66	1	HNYS-015
9	*Chimonobambusa pachystachys* Hsuch et W. P. Zhang	Cizhuzi 刺竹子	alcuq	Acu	Poaceae	Herb	Wild	35	0.85	2	HNYS-017
10	*Crataegus pinnatifida* Bunge	Shanzha 山楂	siqlagaoq siiq alzaol	Xilaguoxiazong	Rosaceae	Tree	Wild and cultivated	25	0.61	1	HNYS-020
11	*Cunninghamia lanceolata* (Lamb.) Hook	Shanmu 杉木	dalpeissaq	Tapeiran	Cupressaceae	Tree	Wild and cultivated	24	0.59	1	HNYS-021
12	*Docynia delavayi* (Franch.) C. K. Schneid	Yunnanduoyi 云南多依	siqpyuqssaq	Sipeiran	Rosaceae	Tree	Wild	34	0.83	2	HNYS-022
13	*Eurya nitida* Korthals	Xichiyeling 细齿叶柃	byuqsulssaq	Bisuran	Pentaphylacaceae	Tree	Wild	34	0.83	2	HNYS-025
14	*Ficus concinna* Miq	Yarong 雅榕	niaolkaolssaq	Niukongran	Moraceae	Tree	Wild	20	0.49	1	HNYS-027
15	*Imperata cylindrica* (L.) Beauv	Baimao 白茅	wuvqjil	Wuji	Poaceae	Herb	Wild	36	0.88	2	HNYS-029
16	*Juncus effusus* L	Dengxincao 灯芯草	jakaol	Jiangkong	Juncaceae	Herb	Wild	30	0.73	1	HNYS-030
17	*Malus pumila* Mill	Pingguo 苹果	piqgao siiq alzaol	Pingguoxiazong	Rosaceae	Tree	Cultivated	18	0.44	1	HNYS-031
18	*Microstegium ciliatum* (Trin.) A. Camus	Gangxiuzhu 刚莠竹	eiqziqmoqzal	Aizimoza	Poaceae	Herb	Wild	30	0.73	1	HNYS-032
19	*Millettia reticulata* Benth	Aidouteng 崖豆藤	aoqhaoldaoqmiqzal	Ehuoduomiza	Fabaceae	Liana	Wild	19	0.46	1	HNYS-033
20	*Molinia japonica* Hack	Ribenmaishicao日本麦氏草	niuqzal zalhaq	Niuzazaha	Poaceae	Herb	Wild	6	0.15	1	HNYS-034
21	*Musa acuminata* var. *sumatrana* (Becc.) Nasution	Yebajiao 野芭蕉	avpavavwuq	Abaawu	Musaceae	Herb	Wild	26	0.63	1	HNYS-035
**22**	*Musa basjoo* Siebold & Zucc. ex Iinuma	Bajiao 芭蕉	pavlqavl	Bajia	Musaceae	Herb	Wild and cultivated	37	0.90	3	HNYS-036
**23**	*Oryza sativa* L	Dao 稻	ceil zaol	Cenzong	Poaceae	Herb	Cultivated	39	0.95	3	HNYS-037
**24**	*Photinia beauverdiana *C.K. Schneid	Zhonghuashinan中华石楠	ziqxoqssaq	Jixueran	Rosaceae	Tree	Wild	7	0.17	1	HNYS-038
**25**	*Phyllostachys sulphurea* (Carr.) A. et C. Riv	Jinzhu 金竹	almol	Amo	Poaceae	Herb	Wild	38	0.93	3	HNYS-039
**26**	*Prunus cerasoides* (D. Don) Sok	Gaopenyingtao 高盆樱桃	yeiqhaqssaq	Yeharan	Rosaceae	Tree	Wild	20	0.49	1	HNYS-040
**27**	*Prunus persica* L	Tao 桃	aqpeil siiq alzaol	Angpeixiazong	Rosaceae	Tree	Cultivated	19	0.46	1	HNYS-041
**28**	*Pueraria montana* var. *lobata* (Willdenow) Maesen & S. M. Almeida ex Sanjappa & Predeep	Gegen 葛根	qilguqzaq	Qiguza	Fabaceae	Liana	Wild and cultivated	7	0.17	1	HNYS-042
**29**	*Pyrus betulifolia* Bunge	Duli 杜梨	aqpeil peilciiv siiq alzaol	Angpeipeizixiazong	Rosaceae	Tree	Wild	20	0.49	1	HNYS-043
**30**	*Pyrus xerophila* Yü	Muli 木梨	aqpeilsiiq	Angpeixi	Rosaceae	Tree	Wild	25	0.61	1	HNYS-044
**31**	*Rhus chinensis* Mill	Yanfumu 盐肤木	siqmassaq	Ximaran	Anacardiaceae	Tree	Wild	40	0.98	4	HNYS-045
**32**	*Rubus ellipticus* var. *obcordatus* (Franch.) Focke	Zaiyangpao 栽秧泡	huvqsil aqgao	Husiaguo	Rosaceae	Shrub	Wild	29	0.71	1	HNYS-046
**33**	*Salix cavaleriei* Levl	Yunnanliu 云南柳	haqsavlolnoq	Hasaenuo	Salicaceae	Tree	Wild	17	0.41	1	HNYS-047
**34**	*Schima argentea* Pritz. ex Diels	Yinmuhe 银木荷	siqsalssaq	Xisaran	Theaceae	Tree	Wild	29	0.71	1	HNYS-048
**35**	*Spatholobus suberectus* Dunn	Mihuadou 密花豆	laqbeilniqzal	Labeinizha	Fabaceae	Liana	Wild	28	0.68	1	HNYS-049
**36**	*Stewartia pteropetiolata* W. C. Cheng	Chibingzijing 翅柄紫茎	noqnilssaq	Nuoniran	Theaceae	Tree	Wild	8	0.20	1	HNYS-050

**Table 3:** Detailed information on these rituals

No.	Ritual name (transliteration of Hani name)	Ritual name	Ritual time	Ritual location	Persons who perform rituals	Reasons for performing rituals and functions of rituals
1	Ahuihuisuohong	Ritual of calling sb’s soul	The timing of the ritual is not fixed; when the host thinks it is necessary	The host of the ritual	One Beima	Someone was bitten by a snake, and the ritual was performed to bring his soul back so that the whole family would be healthy
2	Angmatu	Ritual of worshiping the village god	The first dragon day in February of the lunar calendar lasts five days	Moqiuchang and the sacred forest of the village	One Beima and four Migus	Worship the village gods to seek blessings and protection from the village gods, and pray for the villagers’ good health, prosperity of livestock, and good harvests. They are preparing for spring plowing
3	Boza	Funeral	The timing of funerals is not fixed; they are held whenever someone passes away	Home of the deceased	Several Beimas	May the departed rest in peace, their souls return to their ancestral place, and the living thrives in health and prosperity
4	Dekayaza	Purification ritual	The timing of the ritual is not fixed; when the host thinks it is necessary	The host of the ritual	One Beima	Bees fly to build nests under the eaves. Clean the yard and remove all unclean and unsanitary things in the house
5	Hasaza	Ritual of asking for peace	The first month in the lunar calendar lasts for one day and concludes	The host of the ritual, in the forest	One Beima	Before going out, seek safety, good health, and abundant wealth
6	Huobihuozuo	Ritual of stabilizing the house	The timing of the ritual is not fixed; when the host thinks it is necessary	The host of the ritual	One Beima	Someone dreams that their house has collapsed and performs rituals to stabilize it so that it will not collapse in real life
7	Huoxiza	Xinmijie	The first dragon day in August of the lunar calendar	In the terraced fields, every household	Head of each household	They are grateful to their ancestors and pray for their blessings, abundant crops, prosperous livestock, good health of the villagers, and prosperity of the population
8	Kuzhazha	Farming sacrificial ritual	The 24th day of the sixth month in the lunar calendar lasts three days	Moqiuchang, Moqiu house, Every household	Four Migus	Pray that the crops will be abundant, the livestock will be prosperous, the villagers will be healthy, and the population will be thriving
9	Mulania	Ritual of erecting the stone tablets of merit	The timing of the ritual is not fixed; they are held when a particular family needs to erect the stone tablets of merit	Roadside or village roadside	One Beima	Some women may face difficulties in conceiving, or there may be individuals with health issues in the household. In such cases, constructing stone monuments and platforms is undertaken to accumulate merits through virtuous deeds in hopes of receiving blessings and positive outcomes
10	Ximaganiusa	Disaster relief ritual, drive away swine fever	The first pig day in the seventh month of the lunar calendar	Every household	Head of each household	To eradicate swine fever; all undesirable and unclean elements within the household are expelled
11	Zuoruotu	Ritual of erecting a central pillar	The timing of the ritual is not fixed; they are held when a particular family needs to erect a center pillar	The host of the ritual	One Beima	A central pillar is erected in the house, and all ritual activities in the home revolve around this pillar. It represents the life tree of the family

**Table 4:** Specific applications of ritual plants

Ritual name	Number of plants used	Scientific name of plant	Parts used	How to use ritual plants
1.Ahuihuisuohong (Ritual of calling sb’s soul)	1	*Musa basjoo* Siebold & Zucc. ex Iinuma	Leaf	After reciting the ritual chants, rice and cabbage are wrapped
	2	*Phyllostachys sulphurea* (Carr.) A. et C. Riv	Stem, leaf	Old bamboo is used to make ancestral spirits, and young bamboo is used to make bamboo wine cups
	3	*Rhus chinensis* Mill	Bark	Seal bamboo wine glasses with bark
2.Angmatu (Ritual of worshiping the village god)	1	*Oryza sativa* L	Stem, leaf	Weaving straw rope is called “village gate” by the Hani people
	2	*Rhus chinensis* Mill	Stem	Cut it into nine wooden hammers and nine wooden knives, mark them with burnt charcoal, and hang them on the “village gate” made of straw
3.Boza (Funeral)	1	*Alnus nepalensis* D. Don	Stem	Make “ghost umbrellas”; use them as firewood for the deceased
	2	*Bambusa emeiensis* L. C. Chia & H. L. Fung	Stem	A large bamboo stands at the entrance of the deceased’s home; Beima transforms it into a bamboo tube to guide the spiritual journey
	3	*Chimonobambusa pachystachys* Hsuch et W. P. Zhang	Stem	Preparation of sacrificial altar “Boge”
	4	*Eurya nitida* Korthals	Branch, leaf	The purest tree; crafting “ghost umbrellas”
	5	*Juncus effusus* L	Whole plant	Weave grass mats for the deceased’s coffin
4.Dekayaza (Purification ritual)	1	*Musa basjoo* Siebold & Zucc. ex Iinuma	Leaf	After reciting the ritual words, they wrap rice and vegetables and deliver them outside the house
	2	*Phyllostachys sulphurea* (Carr.) A. et C. Riv	Stem, leaf	Crafting ancestral spirits, hanging chicken feathers and duck feathers
5.Hasaza (Ritual of asking for peace)	1	*Rhus chinensis* Mill	Stem	Carving into the shape of wooden knives to ward off evil spirits
6.Huobihuozuo (Ritual of stabilizing the house)	1	*Imperata cylindrica* (L.) Beauv	Stem, leaf	Placing them on the roof symbolizes preventing the house from leaking during rainfall
	2	*Musa basjoo* Siebold & Zucc. ex Iinuma	Leaf	Placing them on the roof symbolizes preventing the house from leaking during rainfall
	3	*Phyllostachys sulphurea* (Carr.) A. et C. Riv	Stem, leaf	Carving bamboo into strips and placing them on the door and walls
	4	*Salix cavaleriei* Levl	Stem, branch, leaf	Placing them around the house to prevent the house from loosening
7.Huoxiza (Xinmijie Festival)	1	*Oryza sativa* L	Stem, leaf	Suspending them on the central pillar, symbolizing the presence of the true ancestors
8.Kuzhazha (Farming sacrificial ritual)	1	*Acer sinense* Pax	Stem, branch	Securing the “Moqiu” in place
	2	*Alnus nepalensis* D. Don	Stem	Crafting the “Moqiu”
	3	*Amomum tsaoko* Crevost et Lemarie	Stem	Decorating sacrifices. Tying bundles of sticky rice and eggs wrapped in banana leaves
	4	*Bambusa emeiensis* L. C. Chia & H. L. Fung	Stem	Crafting bamboo strips to secure thatched grass on the roof of the Moqiu house
	5	*Castanopsis chinensis* (Sprengel) Hance	Stem	Using three wooden poles as swing frames, one bundle of wooden sticks for constructing the beams on the Moqiu house, securing two wooden sticks for the Moqiu, and utilizing them as Moqiu pillars
	6	*Celtis tetrandra* Roxb	Stem	One swing frame; securing the swing frame
	7	*Chimonobambusa pachystachys* Hsuch et W. P. Zhang	Stem	Weaving a small bamboo raft called “Boge” for the altar inside the Moqiu house
	8	*Docynia delavayi* (Franch.) C. K. Schneid	Whole plant	The sacred tree is worshipped
	9	*Eurya nitida* Korthals	Branch, leaf	After setting up the swing frame, the topmost position is reserved for an inverted placement. The first swing is adorned with green branches of the trees
	10	*Imperata cylindrica* (L.) Beauv	Stem, leaf	The thatched grass lay on the roof of the Moqiu house
	11	*Microstegium ciliatum* (Trin.) A. Camus	Branch, leaf	Offering horse fodder to the celestial horse, the mount of the heavenly deity. Three small bundles, each containing nine stalks, are hung on the Moqiu house
	12	*Millettia reticulata* Benth	Stem	The delicate vines used to connect the swing frames
	13	*Molinia japonica* Hack	Stem, leaf	Securing the swing frames
	14	*Musa acuminata var. sumatrana* (Becc.) Nasution	Leaf	Wrapping sticky rice, eggs, and layering beef on the leaves
	15	*Photinia beauverdiana* C.K. Schneid	Stem	Constructing the swing frames
	16	*Prunus cerasoides* (D. Don) Sok	Stem	Assembling the “Moqiu Pillars”
	17	*Pueraria montana var. lobata* (Willdenow) Maesen & S. M. Almeida ex Sanjappa & Predeep	Stem	Connecting and securing the swing frames together
	18	*Schima argentea* Pritz. ex Diels	Whole plant	The sacred tree is worshipped
	19	*Spatholobus suberectus* Dunn	Stem	Swing; fasten the swing frames; secure the Moqiu structure
	20	*Stewartia pteropetiolata* W. C. Cheng	Stem	The wooden rods were used to construct the beams on the Moqiu house
9.Mulania (Ritual of erecting the stone tablets of merit)	1	*Crataegus pinnatifida* Bunge	Whole plant	Virtue and charity: planted next to various types of stone monuments and platforms to provide shade and coolness for passers-by and to quench their thirst and hunger
	2	*Docynia delavayi* (Franch.) C. K. Schneid	Whole plant	Virtue and charity: planted next to various types of stone monuments and platforms to provide shade and coolness for passers-by and to quench their thirst and hunger
	3	*Malus pumila* Mill	Whole plant	Virtue and charity: planted next to various types of stone monuments and platforms to provide shade and coolness for passers-by and to quench their thirst and hunger
	4	*Prunus persica* L	Whole plant	Virtue and charity: planted next to various types of stone monuments and platforms to provide shade and coolness for passers-by and to quench their thirst and hunger
	5	*Pyrus betulifolia* Bunge	Whole plant	Virtue and charity: planted next to various types of stone monuments and platforms to provide shade and coolness for passers-by and to quench their thirst and hunger
	6	*Pyrus xerophila* Yü	Whole plant	Virtue and charity: planted next to various types of stone monuments and platforms to provide shade and coolness for passers-by and to quench their thirst and hunger
10.Ximaganiusa (Disaster relief ritual, drive away swine fever)	1	*Artemisia caruifolia* Buch.-Ham. ex Roxb	Stem, leaf	Suspending on the door, suspending on the door of pigsty; Cleaning and repelling swine fever from the household
	2	*Capsicum annuum* L	Fruit	Inserting the knife made of *Rhus chinensis* Mill to ward off swine fever. Suspending on the door, suspending on the door of pigsty
	3	*Rhus chinensis* Mill	Stem	Carving tree branches into knife-like shapes and drawing stripes with black charcoal to ward off ghosts and plague spirits. Suspending on the door, suspending on the door of pigsty
	4	*Rubus ellipticus var. obcordatus* (Franch.) Focke	Stem, leaf	Suspending on the door, suspending on the door of pigsty. Cleaning and repelling swine fever from the household
11.Zuoruotu (Ritual of erecting a central pillar)	1	*Castanopsis chinensis* (Sprengel) Hance	Stem	Erecting a central pillar
	2	*Cunninghamia lanceolata* (Lamb.) Hook	Stem	Erecting a central pillar
	3	*Ficus concinna* Miq	Stem, branch, leaf	Erecting a central pillar
	4	*Oryza sativa* L	Stem, leaf	Suspended on the central pillar, representing the true ancestors

**Table 5:** Reasons for using ritual plants

Reason for using ritual plants	Number	Proportion (%)
Biological attribute	38	92.68
Decoration	28	68.29
Eliminate disasters	26	63.41
Communication with ancestors	20	48.78
The expression of the will of the population to reproduce	18	43.90
Prayer for well-being and vision	15	36.59

### Frequency of citation (FC) and relative frequency of citation (RFC)

This index, which does not consider the variable u (use-category), is obtained by dividing the number of informants who mention the use of the species, also known as frequency of citation (FC), by the number of informants participating in the survey (N). Using the same terminology, the numerator can be seen as the summation of the UR (use-report) of all the informants interviewed for the species without considering the use-category [28].

$${RFC}_{s}=\frac{{FC}_{s}}{N}=\frac{\sum_{i={i}_{1}}^{{i}_{N}}{UR}_{i}}{N}$$

For example, *Alnus nepalensis* D. Don was reported as useful by 34 out of 41 informants; hence, RFC_*Alnus nepalensis* D. Don_ = 34/41 = 0.83. This index theoretically varies from 0, when nobody refers to the plant as useful, to 1 in the unlikely case that all the informants would mention the use of the species.

## Results

### Reported ritual plants and rituals

**Fig. 7:**
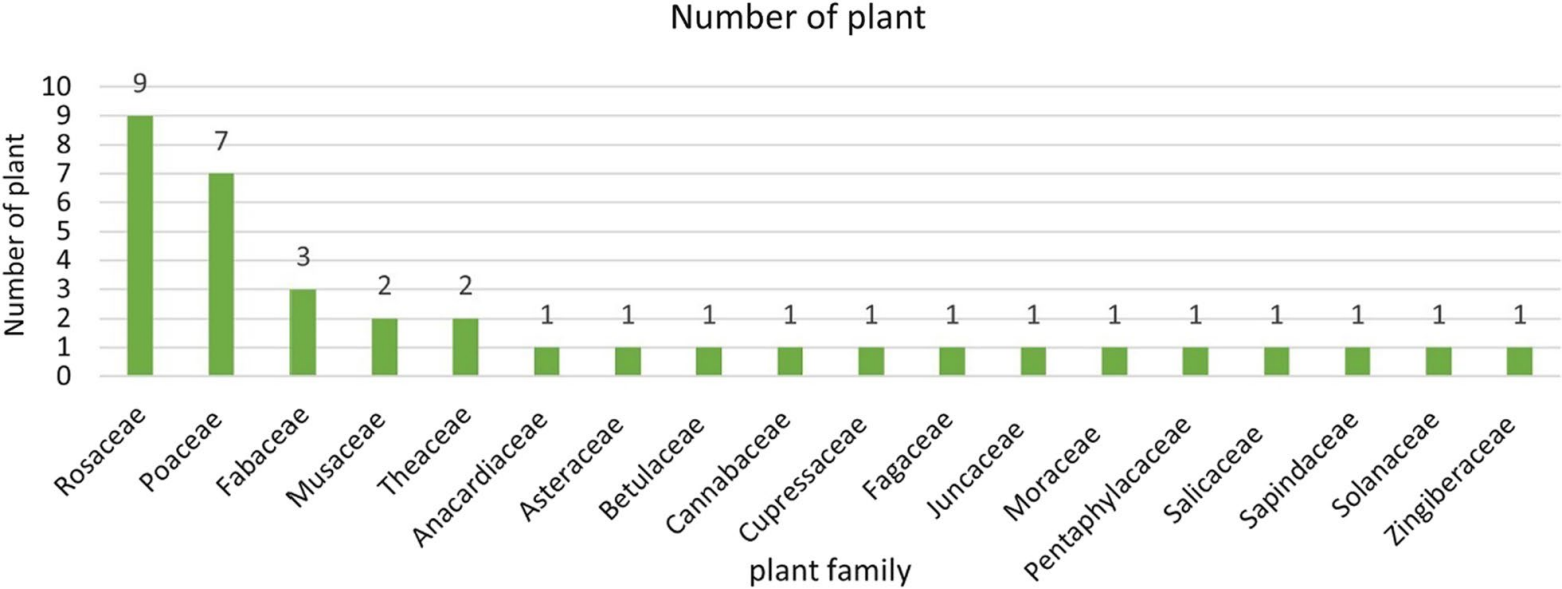
Family of investigated ritual plants

**Fig. 8:**
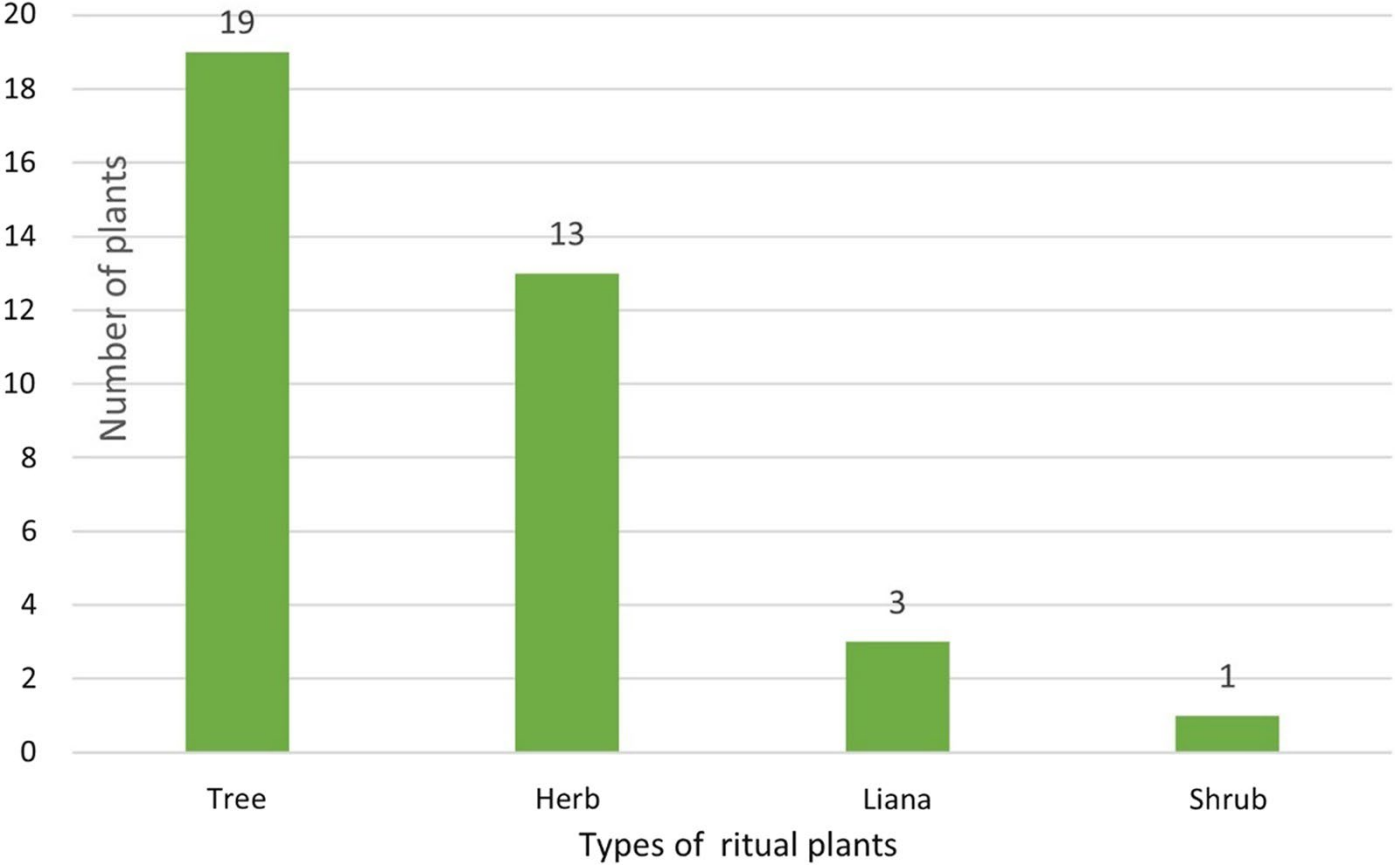
Types of investigated ritual plants

**Fig. 9:**
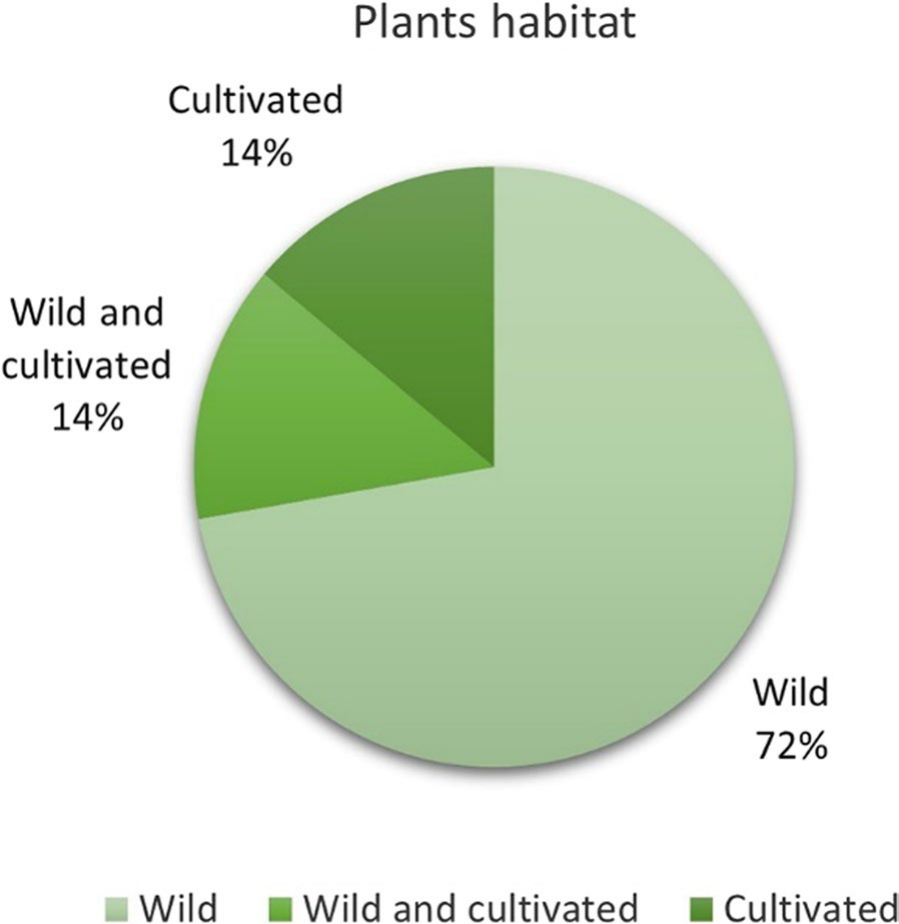
Habitats of investigated plants

We recorded 36 ritual plants, which belong to 18 families (Table 2). Most of the ritual species belong to Rosaceae, Poaceae and Fabaceae families (Fig. 7). The 36 identified ritual plant species were divided into four types: 19 trees, 13 herbs, three lianas, and one shrub (Fig. 8). Most of the ritual plants chosen by Hani were collected from the wild, primarily found in forests, terraced fields, and surrounding villages. Among these plants, ten species (27.78%) are cultivated through human intervention (Fig. 9).

A total of 11 rituals were observed and documented, which involved in the use of the plants mentioned above; these rituals are the Ritual of calling sb’s soul (Ahuihuisuohong), Ritual of worshiping the village god (Angmatu), Funeral(Boza), Purification ritual (Dekayaza), Ritual of asking for peace (Hasaza), Ritual of stabilizing the house (Huobihuozuo), Xinmijie Festival (Huoxiza), Farming sacrificial ritual (Kuzhazha), Ritual of erecting the stone tablets of merit (Mulania), Disaster relief ritual and drive away swine fever (Ximaganiusa), Ritual of erecting a central pillar (Zuoruotu) (Table 3). The 11 rituals are primarily centered around people, crops, and livestock themes. Their overall significance is to pray for the blessings and protection of ancestors and deities and the well-being, cleanliness, and safety of family members. The rituals also seek to ensure prosperity in terms of population growth, bountiful harvests of crops, and thriving livestock (Table 3).

Most of the plants (25 species) were only used in one type of ritual. However, *Rhus chinensis* Mill was selected by the Hani people in four types of rituals. *Phyllostachys sulphurea* (Carr.) A. et C. Riv., *Musa basjoo* Siebold & Zucc. ex Iinuma and *Oryza sativa* L. were selected by the Hani people in three rituals; *Docynia delavayi* (Franch.) C. K. Schneid*, Castanopsis chinensis* (Sprengel) Hance, *Alnus nepalensis* D. Don, *Eurya nitida* Korthals*, Imperata cylindrica* (L.) Beauv, *Bambusa emeiensis* L. C. Chia & H. L. Fung and *Chimonobambusa pachystachys* Hsuch et W. P. Zhang, the seven plants were selected in two rituals (Table 2).

**Fig. 10:**
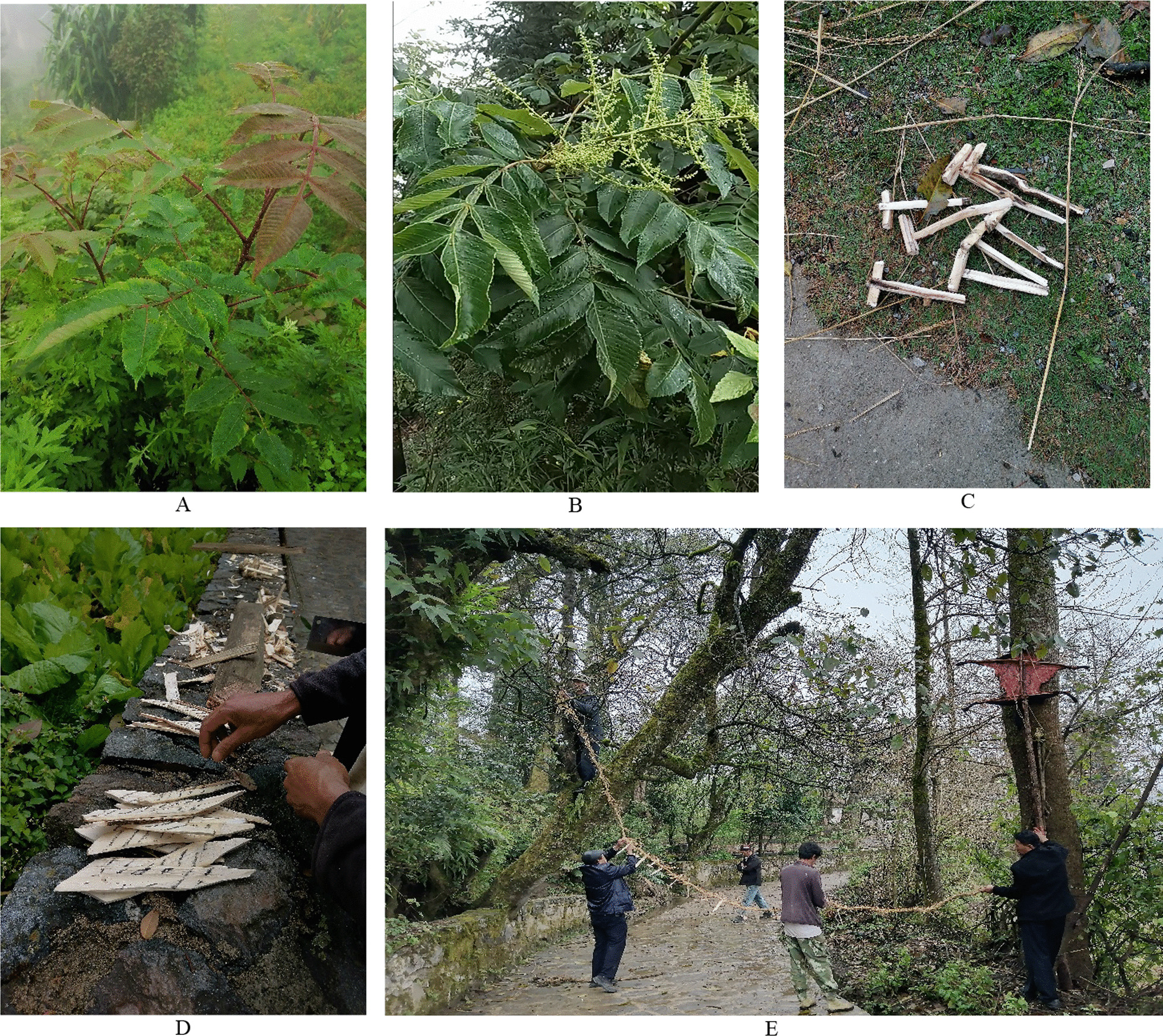
**A** Leaves of *Rhus chinensis* Mill; **B** Flowers of *Rhus chinensis* Mill; **C** Small wooden hammers made by *Rhus chinensis* Mill; **D** Small wooden knives made by *Rhus chinensis* Mill; **E** “Village Gate” hanging small wooden hammers and knives made by *Rhus chinensis* Mill in ritual of worshiping the village god “Angmatu”

According to FC and RFC value, *Rhus chinensis* Mill (Fig. 10), *Oryza sativa* L, *Phyllostachys sulphurea* (Carr.) A. et C. Riv. and *Musa basjoo* Siebold & Zucc. ex Iinuma were the most mentioned species, and the RFC value is 0.98, 0.95, 0.93 and 0.90, respectively (Table 2).

### Application of ritual plants in each ritual

Hani people follow a consistent process for collecting plants in preparation for various traditional ritual practices. Typically, 1–2 days before the commencement of the ritual, they venture into forests, valleys, village surroundings, and terraced fields to gather the required plants. After that, they clean and remove withered branches and yellow leaves of the plants, and then put them in the cleanest area of their houses, where they are not casually stepped upon.

**Fig. 11:**
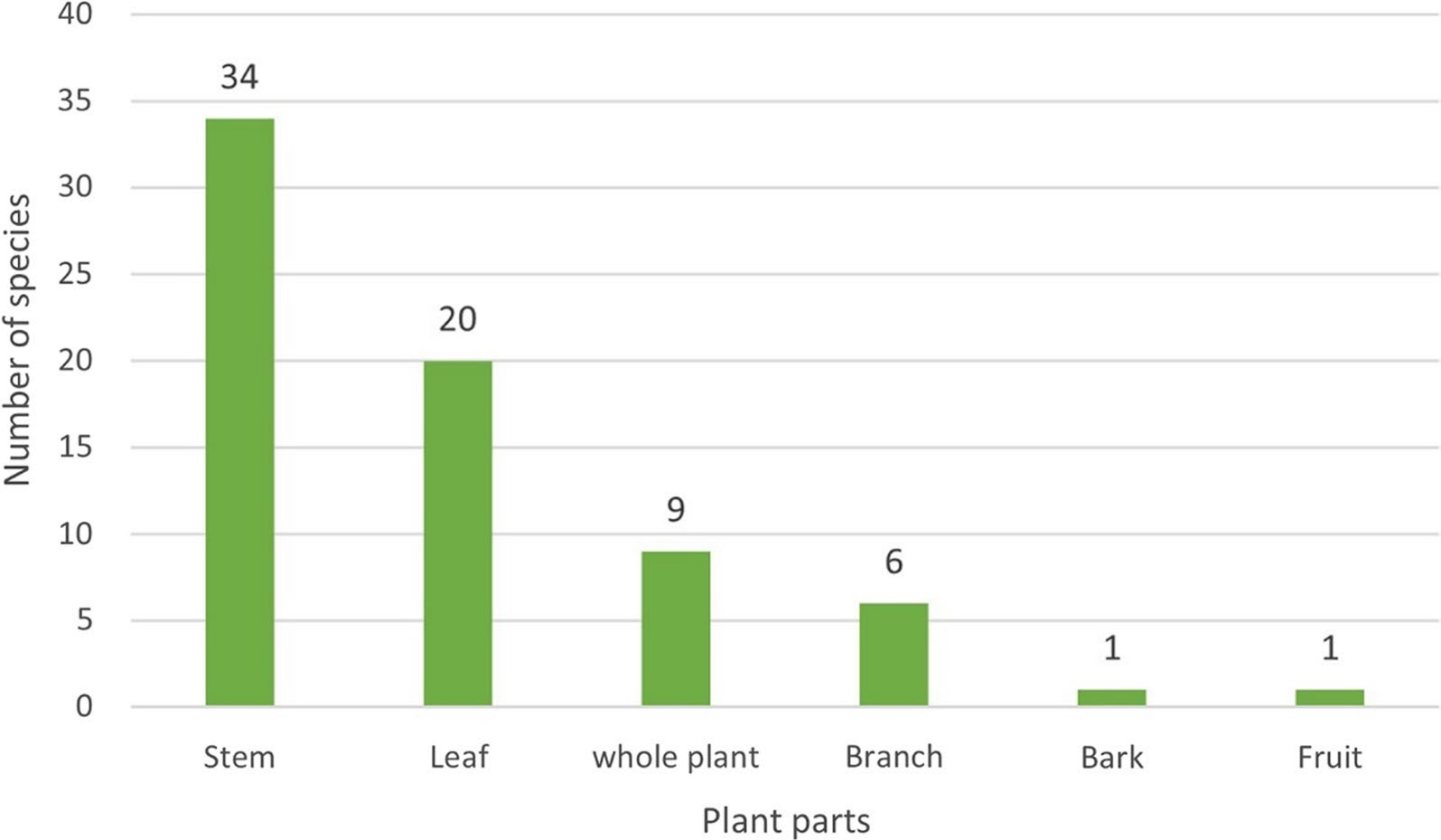
Parts utilized

Hani people utilize various parts of plants, including the whole plant, stem, branch, leaf, fruit, and bark in the 11 types of rituals. Stem is the most frequently employed part (34 species) for ritual use, followed by leaves (20 species), whole plants (9 species), etc. (Fig. 11).

Each plant used in the 11 rituals has specific roles, and even if the same plant is used, its usage varies across different rituals (Table 4). For example, bark and stem of *Rhus chinensis* Mill are used in different ways in different rituals. In general, ritual plants’ primary functions can be decoration, warding off evil spirits, cleansing, seeking peace and safety, seeking fertility and blessings, creating ancestral spirits, and making ritual tools (Table 4).

### Reported reasons for using ritual plants

There were six reasons reported by the informants for the use of ritual plants, including (1) inherent biological attributes of the plants, such as certain morphological features, living habits, habitat, color, and posture; (2) decorative purposes; (3) using specific plants to expel or ward off disasters; (4) responding to the collective historical memory of the Hani people and achieving communication with ancestors; (5) expressing the current population’s reproductive will; (6) praying for well-being and aspirations (Table 5).

Majority of the participants (92.68%) mentioned that the biological characteristics of the plants determine their suitability for ritual purposes. The Hani people selected plants for different rituals mainly based on their characteristics, such as being upright and sturdy, evergreen throughout the seasons, bearing abundant fruits, having varying tree ages, exhibiting strong vitality, and possessing certain physical appearances. These choices reflect the Hani people’s understanding of nature and life and their reverence for ancestors and deities.

## Discussion

### The purpose and significance of Hani people’s performance rituals

In many parts of the world, ritual plants are commonly found. Ritual plants can be used for ritual healing [14], 15, 29] as hallucinogens [30], as incense or decorations for communication with deities [16], or to represent sacred entities such as trees [30]. Hosting rituals often serve multiple objectives, but addressing psychological issues is paramount. Rituals can provide culturally diverse solutions to complex practical problems, achieving multiple desired outcomes. Rituals thrive when they encompass diverse goals [8]. The main themes in the Hani people’s ritual activities is seeking blessings and protection for people, crops, and livestock. The 11 rituals of this study revolve around the above mentioned three themes, with the overall intention of averting disasters and seeking the blessings of deities and ancestors for the prosperity and well-being of the Hani people, abundant and fruitful harvests, and thriving livestock.

The Hani people, whose primary livelihood is rice terrace farming, closely link their ritual performances to their production and daily life. The enduring interest of the Hani people in hosting rituals stems from two main factors. Firstly, agricultural activities serve as a substantial driving force behind rituals. In the harsh ecological environment, peoples’ well-being, crops’ growth, and livestock’s prosperity at various stages all require the blessings of deities. For instance, rituals such as the Angmatu (Ritual of worshiping the village god), the Kuzhazha Festival (Farming sacrificial ritual), and the Huoxiza (Xinmijie Festival) are always synchronized with the rhythm of agricultural production. Secondly, rituals are conducted to communicate and interact with nature. When animals in the natural world pose threats to humans, such as snake bites, or encroach upon human living spaces, such as bees building nests under the eaves, or when natural forces disrupt human habitats, such as dreaming of houses collapsing, the Hani people hold rituals to ward off disasters and evil spirits. The aim is to restore order to the disturbed natural order, achieve reconciliation with nature, and realize harmonious coexistence between humans and nature.

### The significance of plants in Hani people’s rituals

Chinese scholar Zou Hui has documented several ritual plants among the Hani people; however, most have yet to be identified at the species level, and scientific names for these plants are lacking. In Zou Hui’s list of frequently named plants in Hani villages, two of the plants correspond to those recorded in our study (*Alnus nepalensis* D. Don and *Imperata cylindrica* (L.) Beauv.) [31]. The Naxi people also hang *Artemisia caruifolia* Buch.-Ham. ex Roxb on their doors [10], which resembles the usage recorded in our study.

According to our research findings, *Rhus chinensis* Mill, *Oryza sativa* L., *Phyllostachys sulphurea* (Carr.) A. et C. Riv., and *Musa basjoo* Siebold & Zucc. ex Iinuma are frequently mentioned plants. The twigs of *Rhus chinensis* were used in ceremonies of Lawa communities [32]. However, based on our findings, *Rhus chinensis* Mill is utilized in four different rituals, including the ritual of calling sb’s soul, the ritual of worshiping the village god, the ritual of asking for peace, and the purification ritual, with the overall intention of warding off evil and dispelling disasters. *Musa basjoo* Siebold & Zucc. ex Iinuma, *Oryza sativa* L., and *Phyllostachys sulphurea* (Carr.) A. et C. Riv. are used in three different rituals. The selection of *Oryza sativa* L. in rituals is not arbitrary; it must be harvested by the ritual participants from their terraced fields to be used in the rituals. These ritual plants are a constant reminder to the Hani people not to forget their ancestors and history, to respect nature, and to use resources appropriately. For the Hani people, whose primary livelihood relies on rice terrace farming, plants play a significant role in their connection with the natural environment, communication with ancestors, warding off disasters, and praying for peace and happiness.

### Cultural symbolism of ritual plants

Symbolism is a concept wherein something represents another through association, similarity, or custom [33]. Initially, imperceptible entities become perceptible through symbolic forms and can be purposefully utilized by society by manipulating religious specialists [34]. In traditional beliefs, ritual plants serve as spiritual mediators connecting humans with intangible forces [35].

Our research findings indicate that the plants selected for traditional rituals among the Hani people share specific common biological attributes when classified according to their habit as trees, shrubs, vines, and herbs, combined with their uses. Based on existing studies, the most common function of sacred trees is to serve as spiritual abodes [36], while white flowers symbolize purity [37]. Just like the two sacred trees (*Schima argentea* Pritz. ex Diels and *Docynia delavayi* (Franch.) C. K. Schneid.) offered in the Kuzhazha Festival, they are tall, sturdy, evergreen, with dense foliage, white flowers, abundant fruits, and long lifespans. The Moqiu pillar in the Kuzhazha Festival serves as a medium connecting the earth, celestial beings, and humans, representing the axis of the universe [38]. Therefore, a straight and sturdy tree species is chosen for this purpose. The tall and sturdy trees used for constructing swings and the Moqiu are characterized by their complex and durable materials, tall and upright stature, evergreen nature, and resistance to drought and poor soil conditions. Vines are used to tie the swings and reinforce the Moqiu due to their cold and drought resistance, evergreen nature, and excellent flexibility. Four herbaceous plants, known for their strong adaptability to the environment and vigorous vitality, exhibiting excellent toughness and resilience, are used to decorate the Moqiu house. The natural attributes of these plants, such as their straight and sturdy nature, good flexibility, and strong tolerance, symbolize the social attributes of integrity, responsibility, strength, and solidity among the Hani people. Based on a thorough understanding, identification, and grasp of the plants’ biological attributes, the Hani people apply them in ritual practices, endowing them with symbolic meanings and cultural significance. These plants, just like the Hani people, embody the ever-growing vitality and exuberance of life in the cosmic world.

### Psychic effect of ritual plants

Most plants in the natural world have a psychic effect, allowing individuals who utilize them to communicate with their ancestors [39]. In the Hani people, bamboo is considered an auspicious plant, representing the external manifestation of human vitality and serving as a spiritual entity for communication between humans and deities [31]. Our research findings indicate that bamboo holds significant psychic effect within Hani Communities. For example, the *Phyllostachys sulphurea* (Carr.) A. et C. Riv. used in the purification ritual known as “Dekayaza” is considered the soul plant and core symbol of the Hani cosmology. It symbolizes the Hani ancestors and represents a response to the past. Cultural practitioner MYJ “Beima” stated:

“Only with the existence of all things in the world can humans survive. As the old saying goes, the largest creature in the water is the fish and the largest on land is bamboo. Bamboo is the embodiment of our Hani ancestors.”

Each symbolically significant object is associated with some experiential element from real-life encounters. Firstly, from the Hani perspective, bamboo makes ancestral spirits because of its vigorous reproductive power and resilient vitality, aligning with the Hani’s desire for prosperity and flourishing descendants. Bamboo is also long-lasting and easily preserved, meeting the ancestral spirits’ need for permanence. Secondly, places with bamboo are typically associated with abundant water sources, clean water, and a symbol of a well-preserved ecological environment.

The small bamboo raft made from *Chimonobambusa pachystachys* Hsuch et W. P. Zhang, known as “boqgeel” in the Hani language, refers to the Hani sacrificial platform and shrine. Whether the “boqgeel” made during the Kuzhazha Festival or funerals, they are used to house and honor ancestral spirits. This practice is because the Hani people encountered rivers during their migration, and the swift currents made it difficult to cross. Suddenly, they discovered a *Chimonobambusa pachystachys* Hsuch et W. P. Zhang forest (ci zhu) by the river, and they wove the “ci zhu” into rafts, which helped the Hani ancestors successfully cross the river and be saved. In gratitude for the life-saving assistance of the “ci zhu”, the Hani people plant it wherever they go, expressing their gratitude to it at all times. During funerals, the Hani people erect *Bambusa emeiensis* L. C. Chia & H. L. Fung on the left side of the main gate of the deceased’s home. Beima crafts it into bamboo tubes used for reciting scriptures and as decorations on the roof of the Moqiu house during the Kuzhazha Festival. These practices are specific manifestations of the psychic effect of ritual plants.

### Conveying the vision and symbolism of population reproduction through ritual plants

The Hani people often use plants in rituals to convey the auspicious symbolism of strong reproductive power, fertility, robustness, and well-being [31]. For instance, during the village festival called the Kuzhazha Festival, every household collects three clusters of *Microstegium ciliatum* (Trin.) A. Camus, each cluster consists of nine plants, totaling 27, then hung on the houses. This particular grass is favored by important working animals such as oxen and horses in agricultural production. It possesses vigorous vitality and a fast growth cycle, and when consumed by oxen and horses, it fills them with strength, injecting energy and vitality into agricultural production.

In the region under our study, the Hani people, apart from selecting plants for rituals, also convey the vision and symbolism of population reproduction through the cultivation of selected plants. These chosen plants are all edible fruit trees belonging to the Rosaceae family. They can be found throughout the traditional Hani community’s production and living spaces. For instance, during the ritual of erecting the stone tablets of merit, plants are planted to ward off illnesses, seek blessings and accumulate merits, and wish for a prosperous future with many descendants. In the terraced rice fields and pathways of the Hani agricultural area, one can frequently encounter stone platforms where farming laborers rest. These stone platforms are typically accompanied by a big or small tree planted by the villagers. The trees include *Crataegus pinnatifida* Bunge, *Docynia delavayi* (Franch.) C. K. Schneid., *Malus pumila* Mill, *Prunus persica* L., *Pyrus betulifolia* Bunge and *Pyrus xerophila* Yü. These trees provide shade and serve as public resting places for the village community. The construction of these stone steps, which offer a space for communal relaxation, is often initiated by villagers with specific desires. These desires often come from women facing difficulties in conceiving, who build the stone steps and plant fruit-bearing trees in the hope of fertility and to provide refreshment and sustenance to passersby, accumulating merits and blessings while seeking fertility. Another group of individuals with specific desires includes households with sick or weak family members. They construct public resting stone platforms and plant shade trees to pray for the early recovery and well-being of their loved ones. In this process, plants are crucial mediators, establishing a transcendent connection with nature. Therefore, the commonly seen fruit trees in the Hani people’s production and living spaces are not merely plants that provide fruits; they carry and embody the essence of Hani culture. These trees gather their hopes, visions, blessings, and the vital spiritual symbols of vitality.

### The utilization of plants represents a significant traditional ecological knowledge among the Hani people

Based on our research findings, the Hani people have persistent dynamic performance rituals, and the fundamental reason behind this is to maintain harmonious coexistence with nature. Plants from the natural world serve as a medium through which the Hani people effectively communicate with nature, and using plants in rituals represents an essential traditional ecological knowledge among the Hani people. Berkes argues that traditional knowledge systems tend to have a large moral and ethical context; there is no separation between nature and culture [40]. Plants and animals are not recognized solely for their utility; they are considered useful or beneficial because they are first understood [41]. Traditional ecological knowledge reflects local people’s attitudes and ways of life, often embedded in rituals and daily cultural practices [42]. The selection and utilization of plants in Hani ritual practices vividly express the cognitive processes and behavioral choices inherent in their understanding and nurturing of relationships with the world. The Hani people engage in “learning from things” and gain inspiration and shared knowledge from the natural world [43]. They develop an affinity and empathy with animate and inanimate objects, perceiving emotions, recording experiences, and passing them down through generations [43]. The interaction between humans and their environment involves empathetic dialogue and reciprocal reflection with the “non-human” realm. Effective interaction with the natural world requires actively perceiving, understanding, accepting, and contemplating the diversity we encounter. Traditional ecological knowledge is a continuation of culture, wisdom, and adaptation to specific environments. It is formed through observations, practices, and accumulated experiences of the environment, tested over time, and adapted to the requirements of specific locations. Traditional ecological wisdom not only persists in everyday life but also has the potential for activation, enriching the concept of ecological civilization [8]. The survival of traditional ecological wisdom in folk culture can better promote the process of ecological civilization construction by activating the ecological wisdom embedded in tradition.

The world’s significant cultural and ritual practices recognize the critical importance of protecting biodiversity and the natural environment for humanity [44–47]. Cultural diversity and biological diversity are inseparable entities [48]. Culture determines individual or collective utilization and management of natural resources, shapes the environment, and influences Earth’s ecosystems and biodiversity [49]. In many regions of Africa, spiritual beliefs can powerfully serve resource and environmental conservation [50–52]. The respect indigenous peoples have for religious or sacred ecological values plays a role in biodiversity conservation to some extent [53]. Indigenous communities are direct participants in utilizing and preserving local vegetation, and their accumulated traditional cultural and ecological knowledge over generations holds significant importance for biodiversity conservation [54]. Similarly, the cultural traditions, ecological beliefs, sacred groves associated with Hani culture, and reverence and protection of sacred trees enhance biodiversity conservation and the environment. Ritual practices like those of the Hani people are vivid examples of respecting and protecting natural resources and ecological systems.

People of our global village differ not only in their daily occupations and material wealth, but also in the ways in which they view the world around them. This multitude of perceptions is directly related to cultural diversity around the world, a diversity that is rapidly shrinking [40]. Just as in the study area, ritual experts who possess botanical knowledge face the challenge of a lack of successors. Cultural diversity and traditional ecological knowledge are gradually diminishing, and some villages have even disappeared. How to protect and inherit traditional ecological knowledge is a question that requires our thoughtful consideration.

## Conclusions

As a Globally Important Agricultural Heritage System (GIAHS) and World Cultural Landscape Heritage, the Hani people inhabited the Honghe Hani Rice Terraces, who have practiced terrace farming as their primary livelihood for generations. They possess rich traditional ecological knowledge related to the utilization of ritual plants. Our research indicates that many Hani ritual activities are closely associated with plants, most of which are collected from the wild. The use of these plants is deeply rooted in Hani's traditional ecological knowledge and beliefs. The 11 traditional rituals revolve around seeking blessings, peace, disaster prevention, and warding off evil spirits. The 36 plant species used in these rituals have various cultural meanings, such as divination, ancestral symbolism, exorcism, deterrence, purification, auspiciousness, peace, and fertility. Through repeated enactment of rituals, the Hani people seek blessings and avoid misfortunes, finding solace and tranquility. The Hani people have a comprehensive understanding of plants, protect plants, and utilize plants. Their reverence and protection of nature, respect for life, gratitude towards ancestors, and seeking blessings and disaster prevention for their families, crops, and livestock are all reflected in these rituals and their utilization of plants.

The Hani people showcase their agricultural culture in the Honghe Hani Rice Terraces through plant-based ritual performances. The close association between plants and Hani culture has given rise to various local knowledge systems passed down through generations. Studying rituals and ritual plants in the core area of the Hani Rice Terraces is of great significance for protecting the Hani terrace farming culture. This research fills the gap in the investigation of ritual plant knowledge in the Honghe Hani Rice Terrace region. It provides a foundation and reference for biodiversity conservation and sustainable development in the GIAHS.

## Data Availability

All data generated or analyzed during this study are included in this published article.

## Abbreviations

FAO: Food and Agriculture Organization
FC: Frequency of Citation
GIAHS: Globally Important Agricultural Heritage System
HNYS: Haniyishi
RFC: Relative Frequency of Citation (RFC)
N: Number of informants participating in the survey
UNESCO: United Nations Educational, Scientific and Cultural Organization
UR: Use report
